# ‘De Novo’ Psychosis following anterior temporal lobectomy: A case report

**DOI:** 10.1192/j.eurpsy.2024.1543

**Published:** 2024-08-27

**Authors:** S. Yoldas, I. Yıldırım, N. G. Usta Sağlam, Ç. Özkara

**Affiliations:** ^1^Psychiatry; ^2^Neurology, Istanbul University-Cerrahpasa, Cerrahpasa Faculty of Medicine, Istanbul, Türkiye

## Abstract

**Introduction:**

Surgical treatments for people living with epilepsy have the potential to provide patients with an opportunity to achieve relief from seizures, thus improving their quality of life, but they are not free of complications. The psychiatric consequences are a significant concern because of the potential risks; however, psychotic illnesses have not received adequate research compared to anxiety and depression.

**Objectives:**

To better identify the psychiatric side effects that can develop following epilepsy surgery, especially psychosis, and to take preventive measures to mitigate its occurrence.

**Methods:**

Presentation of a patient’s case and reviewing existing literature regarding de novo psychosis following epileptic surgery.

**Results:**

The case of interest is a 31-year-old male patient who, or his relatives, has had no history of psychiatric disorders. From age 21, the patient had focal to bilateral seizures, which were preceded by olfactory auras and could occur up to 4-5 times a week and was then diagnosed with epilepsy. In June 2021, the patient underwent a right anterior temporal lobectomy for his medically resistant seizures after a presurgical evaluation and had a notable decrease in the number of seizures, occurring only during periods of sleep every six months. In the fourth month following the operation, the patient began experiencing auditory hallucinations characterized by negative and judgmental voices. After that, he engaged in an aggressive act by holding a knife and assaulting another person in a public area. He was admitted to an inpatient psychiatry service for 12 days with a diagnosis of a psychotic episode. His symptoms significantly improved, and he was discharged with paliperidone 6 mg/daily treatment. After five months, he discontinued the medication, subsequently experiencing a recurrence of auditory hallucinations and aggression. The patient was admitted to the inpatient psychiatric clinic in June 2022 as a result of experiencing paranoid delusions and engaging in a suicide attempt by self-inflicted wrist laceration using a razor blade, which was consistent with the patient’s delusional beliefs. Following 13 days of hospitalization, he was discharged with amisulpride 800 mg/daily in addition to his antiepileptic treatment. After 15 months of discharge, he showed no signs of active psychotic features, and his functioning was moderate to good.

**Conclusions:**

Current research and reporting of psychiatric outcomes are limited, and the predictive factors and prognosis of psychiatric symptoms in these patients remain obscure. Long-term follow-up is crucial, especially considering the possibility of psychiatric symptoms developing in the months following surgery, as demonstrated by the current case. In addition, preoperative and postoperative assessments may facilitate the management of psychiatric symptoms.

**Disclosure of Interest:**

None Declared

